# Comprehensive analysis of skeletal muscle- and bone-derived mesenchymal stem/stromal cells in patients with osteoarthritis and femoral neck fracture

**DOI:** 10.1186/s13287-020-01657-z

**Published:** 2020-04-03

**Authors:** Klemen Čamernik, Anže Mihelič, Rene Mihalič, Gregor Haring, Simon Herman, Darja Marolt Presen, Andrej Janež, Rihard Trebše, Janja Marc, Janja Zupan

**Affiliations:** 1 University of Ljubljana, Faculty of Pharmacy, Chair of Clinical Biochemistry, Askerceva 7, 1000 Ljubljana, Slovenia; 2 Valdoltra Orthopaedic Hospital, Jadranska 31, SI-6280 Ankaran, Slovenia; 3 University of Ljubljana, Faculty of Medicine, Institute of Forensic Medicine, Korytkova 2, 1000 Ljubljana, Slovenia; 4 Clinical Department of Traumatology, University Medical Centre, Zaloska cesta 7, 1000 Ljubljana, Slovenia; 5 Ludwig Boltzmann Institute for Experimental and Clinical Traumatology, AUVA Research Center, Austrian Cluster for Tissue Regeneration, Donaueschingenstrasse 13, A-1200 Vienna, Austria; 6 Department of Endocrinology, Diabetes and Metabolic Diseases, University Medical Centre, Zaloska cesta 2, 1000 Ljubljana, Slovenia

**Keywords:** Mesenchymal stem/stromal cells, Skeletal muscle, Trabecular bone, Osteoarthritis, Osteoporosis, Controls, Leptin receptor

## Abstract

**Background:**

Mesenchymal stem/stromal cells (MSCs) can replenish the aged cells of the musculoskeletal system in adult life. Stem cell exhaustion and decrease in their regenerative potential have been suggested to be hallmarks of aging. Here, we investigated whether muscle- and bone-derived MSCs of patients with osteoarthritis and osteoporosis are affected by this exhaustion, compared to healthy donors.

**Methods:**

Patients with primary osteoarthritis, femoral neck fractures due to osteoporosis, and healthy donors (controls) were included. MSCs were isolated from the skeletal muscle and subchondral bone from each patient and compared using ex vivo and in vitro analyses, including immunophenotyping, colony-forming unit fibroblast assays, growth kinetics, cell senescence, multilineage potential, and MSC marker gene expression profiling.

**Results:**

Freshly isolated cells from muscle from patients with osteoarthritis showed a lower proportion of CD45/CD19/CD14/CD34-negative cells compared to patients with osteoporosis and healthy donors. Freshly isolated muscle cells from patients with osteoarthritis and osteoporosis also showed higher clonogenicity compared to healthy donors. MSCs from both tissues of osteoarthritis patients showed significantly reduced osteogenesis and MSCs from the bone also reduced adipogenesis. Chondrogenic pellet diameter was reduced in bone-derived MSCs from both patient groups compared to healthy donors. A significant positive correlation was observed between adipogenesis and *CD271* expression in muscle-derived MSCs. CD73 was significantly lower in bone-derived MSCs from osteoarthritis patients, compared to osteoporosis patients. Gene expression profiling showed significantly lower expression of MSC marker gene leptin receptor, *LEPR*, previously identified as the major source of the bone and adipocytes in the adult bone marrow, in bone-derived MSCs from patients with osteoarthritis in comparison with osteoporotic patients and healthy donors.

**Conclusions:**

Our results show deficient ex vivo and in vitro properties of both skeletal muscle- and bone-derived MSCs in osteoarthritis and osteoporosis patients, compared to healthy donors. In bone-derived MSCs from patients with osteoarthritis, we also identified a lower expression of the leptin receptor, a marker of MSCs that present a major source of MSCs in the adult bone marrow. This suggests that exhaustion of skeletal muscle- and bone-derived MSCs is a hallmark of osteoarthritis and osteoporosis, which defines the need for further clinical trials of stem cell transplantation in these patients.

## Background

Mesenchymal stem cells (MSCs) have the capacity to regenerate connective tissues following trauma or injury, such as the cartilage, bone, and muscle, and to replenish worn-out tissues and senescent cells [1]. Their reparative properties have been most commonly demonstrated in experimental animal models of cartilage injury [2] and bone fracture [3], while their roles in the pathophysiology of degenerative disorders such as osteoarthritis (OA) and osteoporosis (OP) in humans is not well established. Osteoarthritis Research Society International has indicated OA as a “serious disease,” with the global prevalence of hip and knee OA approaching 5%, which is projected to increase as the population ages [4]. OA manifests first as molecular disorganization, as abnormal joint-tissue metabolism, followed by anatomic, and/or physiological disorganization, such as cartilage degradation, bone remodeling, osteophyte formation, joint inflammation, and loss of normal joint function [4].

Osteoporosis affects over 200 million people worldwide, and both men and women are equally susceptible to developing the age-related form of this chronic disease. In OP, the structure of the bone is lost, which leaves it thinner and more susceptible to fractures. Low-energy fractures of the hip are the most common consequence, which leads to a significant lack of mobility and increased morbidity [5, 6].

Both OA and OP are age-related, and stem cell exhaustion and decreases in their regenerative potential have been defined as one of the hallmarks of aging [7]. Previous studies have shown reduced chondrogenic potential of bone marrow-derived MSCs, with further other alterations to these cells that are implicated in the emergence of OA [8, 9]. OP, on the other hand, has been defined as “obesity of the bone,” as bone marrow-derived MSCs are redirected from osteogenesis to adipogenesis [5, 6].

Age-related OP is commonly accompanied by sarcopenia, a condition that is characterized by muscle weakness, muscle fiber atrophy, and adipose tissue infiltration into muscle [10, 11]. Although the precise cause of sarcopenia remains unknown, it has been proposed that a combination of factors are involved, including systemic inflammation, increased oxidative stress, and changes in skeletal muscle stem cell populations [12–14].

The majority of stem cell therapies currently used in clinical practice is for chondral lesions of major joints and for OA, while stem cell therapies for OP are still in their infancy, due to the lack of human studies [15]. Recent studies in mice, however, have brought new hope for OP patients. These have shown that systemic injections of minimally expanded exogenous MSCs in a mouse model of human age-related OP can markedly increase bone formation and restore the bone microarchitecture [16].

The bone marrow is the most common source for these cell therapies. However, based on previous findings, the bone marrow might not be ideal, as their MSCs can be affected by the concomitant disorder; in particular OA or OP [5, 8, 9]. The skeletal muscle, on the other hand, is an untapped reservoir of MSCs [17]. It is not yet known in OA and OP if the regenerating capacities of muscle-resident MSCs are preserved despite the ongoing degeneration of the bone or cartilage.

To determine whether systemic degenerative disorders of the cartilage and bone, i.e., OA and OP, affect the pool and features of muscle- and bone-derived MSCs, we performed a comprehensive analysis of MSCs derived from the skeletal muscle and trabecular bone in patients with primary OA and low-energy femoral neck fractures (OP) and compared these to those of healthy controls. Our data show that these MSCs from both muscle and bone are affected by these concomitant degenerative disorders, further defining the need for future clinical trials to better evaluate stem cell therapies used in patients suffering from these two musculoskeletal disorders.

## Methods

### Donor inclusion and bone sampling

Patients undergoing routine total hip arthroplasty at the Valdoltra Orthopedic Hospital (OA patients) and patients undergoing partial hip arthroplasty following femoral neck fractures at the Clinical Department of Traumatology (OP patients) were included in this study. Hip osteoarthritis and femoral neck fractures were diagnosed by clinical examination and X-rays. Donors who were undergoing routine autopsies at the Institute of Forensic Medicine were also included in the study (controls; time to autopsy, 4–45 h; mean, 23 h). The exclusion criteria included a history of inflammatory arthritis, metastatic cancer, and disorders that affect the bone.

Approval for the study was obtained from the National Medical Ethics Committee of the Republic of Slovenia (reference numbers: 0120-523/2016-2, KME 45/10/16, 0120-523/2016/11). Written informed consent to participate in this study was obtained from all of the patients. The baseline characteristics for all donors are provided in Table 1.

**Table 1:** Baseline characteristics of the donors included in this study

Donor group	***n***	Age (years)			Female/male	Body mass index
		Mean	Minimum	Maximum		(kg/m^**2**^)
Osteoarthritis	10	72.7**	46	84	7/3	28.1
Femoral neck fracture	10	80.6**	75	91	8/2	25.2
Controls	11	45.1	28	65	4/7	27.9

**Osteoarthritis versus controls *p* = 0.001; femoral neck fracture versus controls *p* = 0.009

The skeletal muscle and trabecular bone were harvested from each patient and from all of the donor groups at the same anatomical site: *gluteus medius* and femoral head, as described previously [18]. Both biopsy samples were immediately placed into growth medium: low-glucose Dulbecco’s modified Eagle’s medium (DMEM; Biowest), with 10% fetal bovine serum (Gibco), 2-mM l-glutamine (Biowest), and 2% antibiotics/antimycotics (100× stock: 8.5 g/L NaCl, 0.025 g/L amphotericin B, 6.028 g/L penicillin G, sodium salt, 10 g/L streptomycin sulfate; all Biowest).

### Cell isolation

**Fig. 1:**
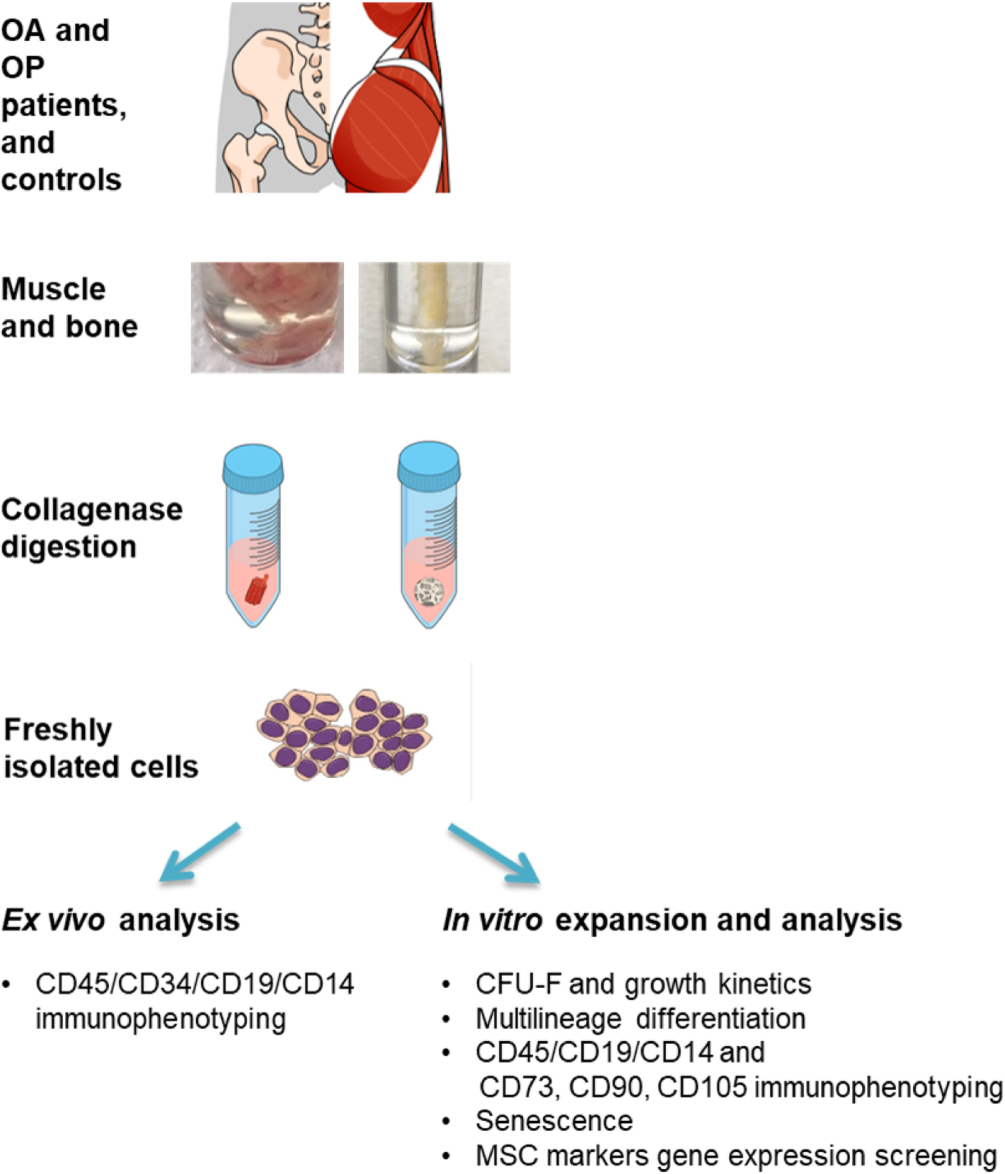
Study design, donor groups, and tissue samples. The skeletal muscle and trabecular bone were collected from the hip joint of all donors. The cells were isolated from both of the tissues using collagenase digestion. An aliquot of freshly isolated cells was used for ex vivo analysis to determine cell viability and the mesenchymal fraction, using flow cytometry. The rest of the freshly isolated cells were culture-expanded to perform in vitro analyses, including the colony-forming unit fibroblast assay, growth kinetics, osteogenic, chondrogenic and adipogenic differentiation, immunophenotyping, senescence, and gene expression of several MSC markers

Primary MSCs were isolated from the muscle and bone biopsies following previously published protocols [19, 20]. Briefly, the muscle and bone biopsies were digested in 1 mg/mL collagenase (Roche) at 37 °C for 1 h and 3 h, respectively. The digested tissue suspension was filtered through a 70-μm nylon strainer (Corning). An aliquot of freshly isolated cells was used for immunophenotyping (ex vivo analysis). The rest of the freshly isolated cells from the muscle were culture-expanded (in vitro analysis). Cells from the muscle were seeded in low-glucose DMEM (Biowest) with 2-mM l-glutamine, and 2% antibiotics/antimycotics (100× stock: 8.5 g/L NaCl, 0.025 g/L amphotericin B, 6.028 g/L penicillin G, sodium salt, 10 g/L streptomycin sulfate; all Biowest). Cells from the bone were seeded in MSC Expansion Medium (Kit XF, human; Miltenyi Biotec) supplemented with 2% penicillin and streptomycin, and 1% glutamine (all Biowest). All of the cultures were maintained at 37 °C in a 5%/5% humidified CO_2_/O_2_ atmosphere. The study design and analyses are summarized in Fig. 1.

### Immunophenotyping

Flow cytometry was performed on an aliquot of both freshly isolated cells and on the culture-expanded MSCs between passages 1 and 5 (p1–p5), as described previously [18, 21]. Freshly isolated cells were tested for CD45, CD19, CD14, and CD34 (mesenchymal fraction) using monoclonal antibodies 2D, SJ25C1, 61D3 (all ThermoFisher Scientific), and AC136 (Miltenyi Biotec), respectively. The culture-expanded MSCs were immunophenotyped using the following antibodies: anti-CD105 MEM-226 FITC (ThermoFisher Scientific), anti-CD90 DG3 FITC, and anti-CD73 AD2 APC (Miltenyi Biotec). The fixable viability dye eFluor 780 (ThermoFisher Scientific) was used to determine cell viability. Flow cytometry was performed using an Attune NxT instrument (ThermoFisher Scientific).

### Colony-forming unit fibroblast assay

The colony-forming unit fibroblast (CFU-F) assay was performed at p0 and p1, as described previously [18]. Briefly, the CFU-F assay data at p0 were calculated as methyl violet-positive colonies per viable MSCs counted after p0 (%). The CFU-F assay data at p1 were calculated as methyl violet-positive colonies per seeded MSCs at p1 (%).

### Cell growth kinetics

Cell-growth kinetics were assessed as described previously [20]. Briefly, the cells were seeded at p1 to p3 as four replicates in 12-well plates at 5000 to 10,000 cells/cm^2^ and counted after 48, 96, 168, and 216 h. Growth curves were constructed, and growth rates and doubling times were calculated from the exponential phases of the growth curves.

### Senescence analysis

Senescence analysis was assessed as described previously [18]. Briefly, the cells were seeded as three to five replicates in 12-well plates at 5000 cells/cm^2^. After 2 to 3 days, when the cultures had reached ~ 30% confluence, senescence was evaluated using senescence β-galactosidase staining kits (Cell Signaling Technology), following the manufacturer instructions. The wells were imaged under a microscope (Primovert) mounted with a digital camera (AxioCam ICc5; Zeiss). The numbers of senescent cells and the total numbers of cells were counted independently by two investigators. Senescence was expressed as the fraction of senescence-associated β-galactosidase-positive cells in the total number of cells.

### Multilineage differentiation

Multilineage differentiation was performed as described previously [18]. Briefly, for adipogenesis and osteogenesis, the cells were seeded as four replicates in 24-well plates at a density of 25,000 cells/cm^2^. Two replicates were used for histological assessment and two replicates for RNA isolation and gene expression analysis, for each control and treated group. The adipogenic media consisted of growth medium supplemented with 500-nM dexamethasone, 10-μM indomethacin, 50-μM iso-butylmethyl xanthine, and 10-μg/mL insulin (all Sigma). The osteogenic medium consisted of growth medium supplemented with 5-mM β-glycerophosphate, 100-nM dexamethasone, and 50-mg/mL ascorbic acid-2-phosphate (all Sigma). Adipogenic differentiation was assessed using Oil Red O (Sigma) histochemistry. The adipogenic efficiency was expressed as the number of Oil Red O-positive adipocytes per number of cells originally seeded (%). Osteogenesis was assessed using Alizarin Red S (Sigma) histochemistry, and the Alizarin Red S concentration was quantified as described previously [18]. For chondrogenesis, 150,000 cells were used to form a chondrogenic pellet. Chondrogenic media consisted of high-glucose DMEM (Biowest), 100-nM dexamethasone (Sigma), 1% insulin-transferrin-selenium (Sigma), 50-mg/mL ascorbic acid-2-phosphate (Sigma), 2% antibiotic/antimycotic (100× stock; Biowest), and 10 ng/mL transforming growth factor (TGF)-β1 (ThermoFisher Scientific). Chondrogenesis was assessed using toluidine blue (Sigma) staining and immunofluorescence for collagen type II (Col2). The chondrogenic potential was evaluated as positive toluidine blue or Col2 staining, and the toluidine blue-stained sections were also evaluated using the Bern score [22]. The chondrogenic cell pellet diameters were determined using the ImageJ software [23].

### RNA isolation and gene expression profiling

Total RNA was isolated from the cells subjected to adipogenesis and osteogenesis, as well as during MSC culture expansion. Gene expression profiling was performed as described previously [18]. Gene expression analysis was performed according to the Minimum Information for Publication of Quantitative Real-Time PCR Experiment guidelines [24]. Briefly, quantitative (q)PCR was performed using 5× HOT FIREPol EvaGreen qPCR Supermix (Solis BioDyne) and gene-specific primers (Macrogen, Sigma-Aldrich). The sequences of the primers used are provided in Supplemental Table 1. Gene expression was measured using a LightCycler 480 II (Roche). All of the data were normalized to glyceraldehyde 3-phosphate dehydrogenase.

### Statistical analysis

Shapiro-Wilk tests were used to test the normality of the distributions of the data. To compare age, body mass index (BMI), and the female to male ratio between the patients with osteoarthritis (OA), osteoporosis (OP), and the controls, two-way ANOVA with Bonferroni corrections for multiple testing was used. Since age proved to be statistically different between the tested group of patients (Table 1), age was used as a covariate when testing the differences between three groups of donors using the general linear model (GLM) and a Bonferroni post hoc. Hence, the differences reported in this study are the result of disease and not age-dependent. The statistical analyses were performed using IBM SPSS Statistics version 25 and GraphPad Prism, version 6. *P* values < 0.05 were considered as statistically significant. The figures were created using Mind the Graph.

## Results

### Patients with osteoarthritis and osteoporosis have lower proportions of MSCs

**Fig. 2:**
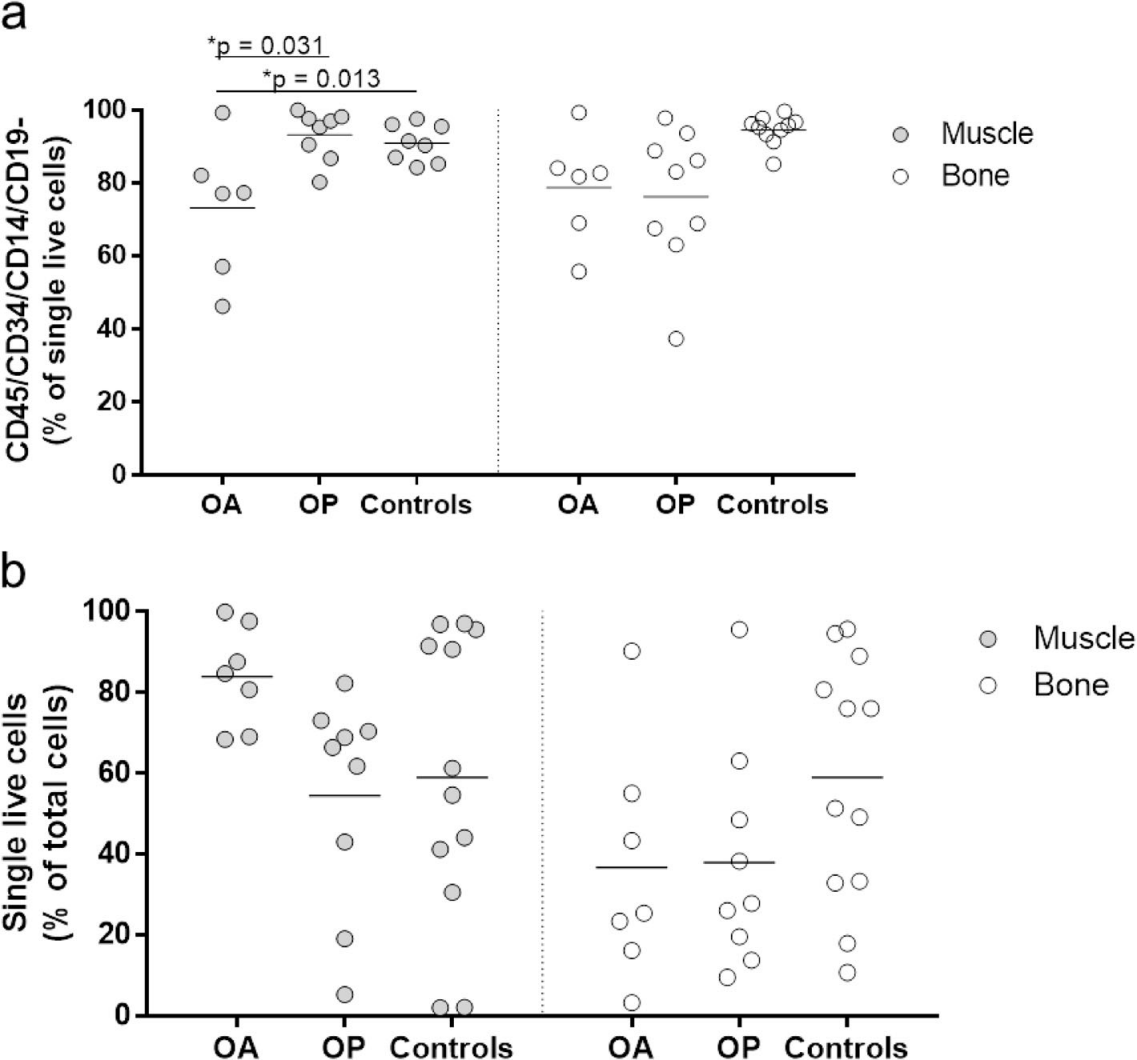
Ex vivo analysis of the freshly isolated cells before in vitro culture expansion. An aliquot of freshly isolated cells was used to determine the proportion of the mesenchymal fraction, as defined as the CD45/CD34/CD14/CD19-negative single live cells (**a**), and to determine the viability of the cells after collagenase digestion (**b**). The proportion of CD45/CD34/CD14/CD19-negative single live cells was significantly lower in OA patients compared to OP patients and controls (73.2% vs. 93.2%, 91.0%; *p* = 0.031, 0.013, respectively), while no differences between the tested groups were observed for the bone-derived MSCs (**a**). The viabilities of the freshly isolated cells were similar between these groups (**b**)

The proportion of the mesenchymal fraction defined as the CD45/CD34/CD14/CD19-negative cells showed significant differences for the muscle-derived MSCs (Fig. 2a). The proportion of these cells was significantly lower in OA patients compared to OP patients and controls (73.2% vs. 93.2%, 91.0%; *p* = 0.031, 0.013, respectively), while no differences between the tested groups were observed for the bone-derived MSCs (Fig. 2a). The viabilities of the freshly isolated cells were similar between these groups (Fig. 2b).

### Freshly isolated muscle-derived MSCs from patients have higher clonogenicity compared to controls

**Fig. 3:**
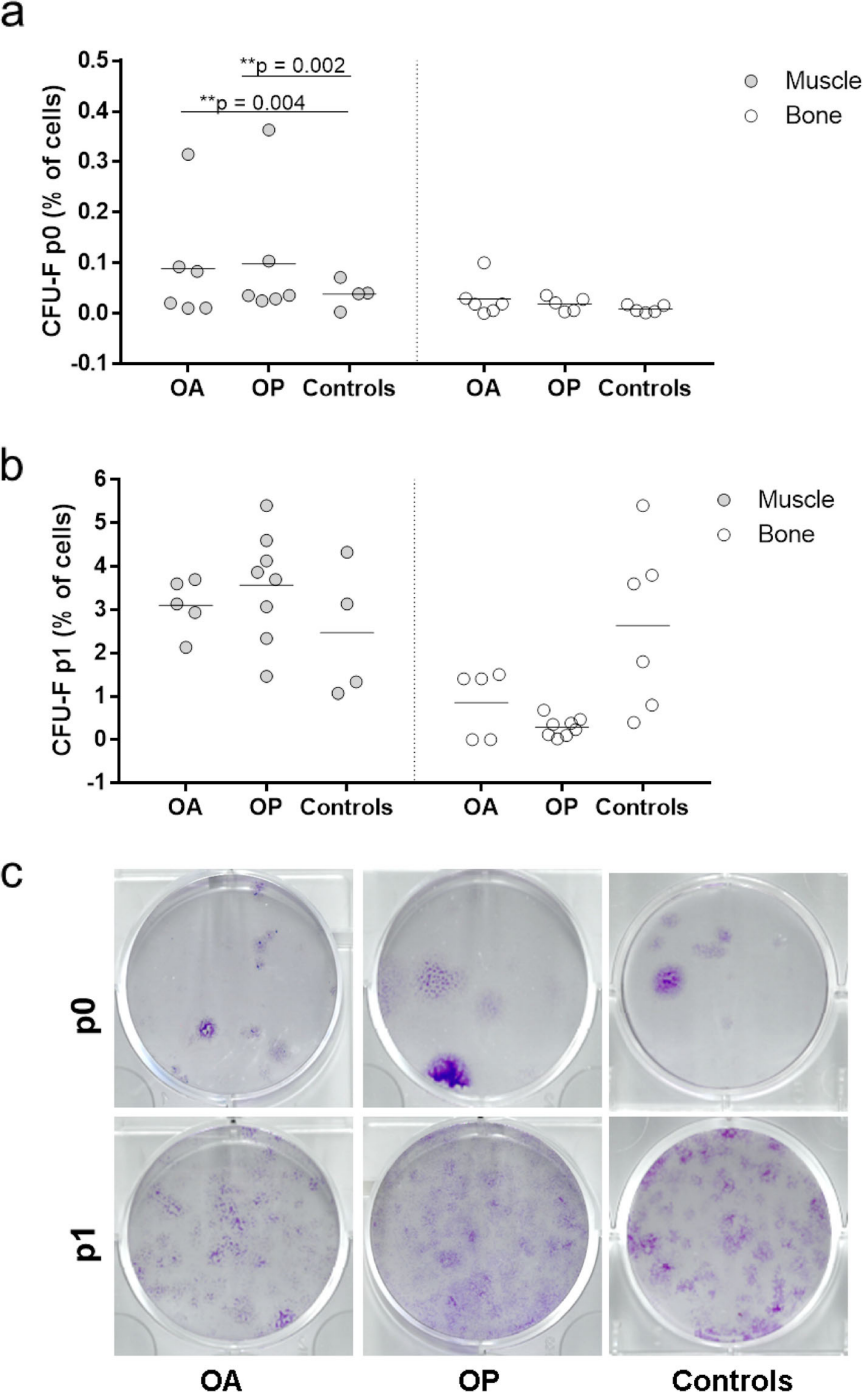
Colony-forming unit fibroblast assay. Cells were plated to perform the colony-forming unit fibroblast assay (CFU-F) at p0 (**a**) and at p1 (**b**). Muscle-derived cells plated at p0 from OA and OP patients showed a significantly higher proportion of cells forming colonies than those from controls (0.0886 and 0.0985% vs. 0.0383%, *p* = 0.004, 0.002, respectively) (**a**). CFU-F assays at p1 did not show any significant differences between the donor groups p1 (**b**). No significant differences were found for bone-derived MSCs (**a**, **b**). Representative images of wells of methyl violet-stained colonies formed after p0 and p1 for muscle-derived cells from OA and OP patients and controls (**c**)

Significant differences were observed in the CFU-F assays at p0 between the muscle MSCs from OA and OP patients and controls (Fig. 3a, c). Muscle-derived cells plated at p0 from OA and OP patients showed a significantly higher proportion of cells forming colonies than those from controls (0.0886 and 0.0985% vs. 0.0383%, *p* = 0.004, 0.002, respectively). However, the CFU-F assays at p1 did not show any significant differences between the donor groups (Fig. 3b, c). No significant differences were found for bone-derived MSCs (Fig. 3).

### Muscle- and bone-derived MSCs from all donor groups show similar culture expansion properties

**Fig. 4:**
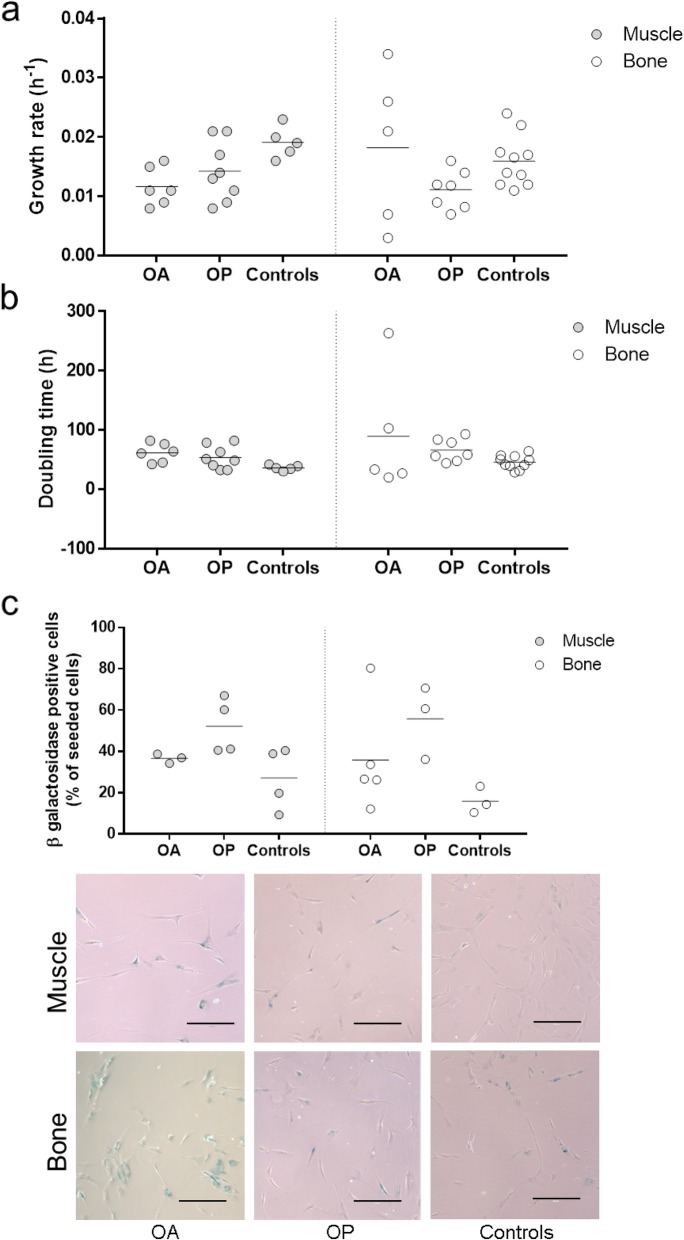
Growth kinetics and senescence. The cells were further culture-expanded after the CFU-F assays at p0 and maintained in culture to determine the growth kinetics, including growth rate (**a**) and doubling time (**b**). The rate of senescence determined by the β-galactosidase staining was determined at p3/p4 for the muscle and bone-derived MSCs of all three donor groups (**c**)

The culture-expanded MSCs across the two patient groups also showed similar growth rates (Fig. 4a) and doubling times (Fig. 4b). Furthermore, they also showed similar fibroblast-like morphologies and similar proportions of senescent β-galactosidase-positive cells (Fig. 4c).

### Adipogenesis of bone-derived MSCs is decreased in osteoarthritis and significantly correlates with CD271 expression in skeletal muscle MSCs

**Fig. 5:**
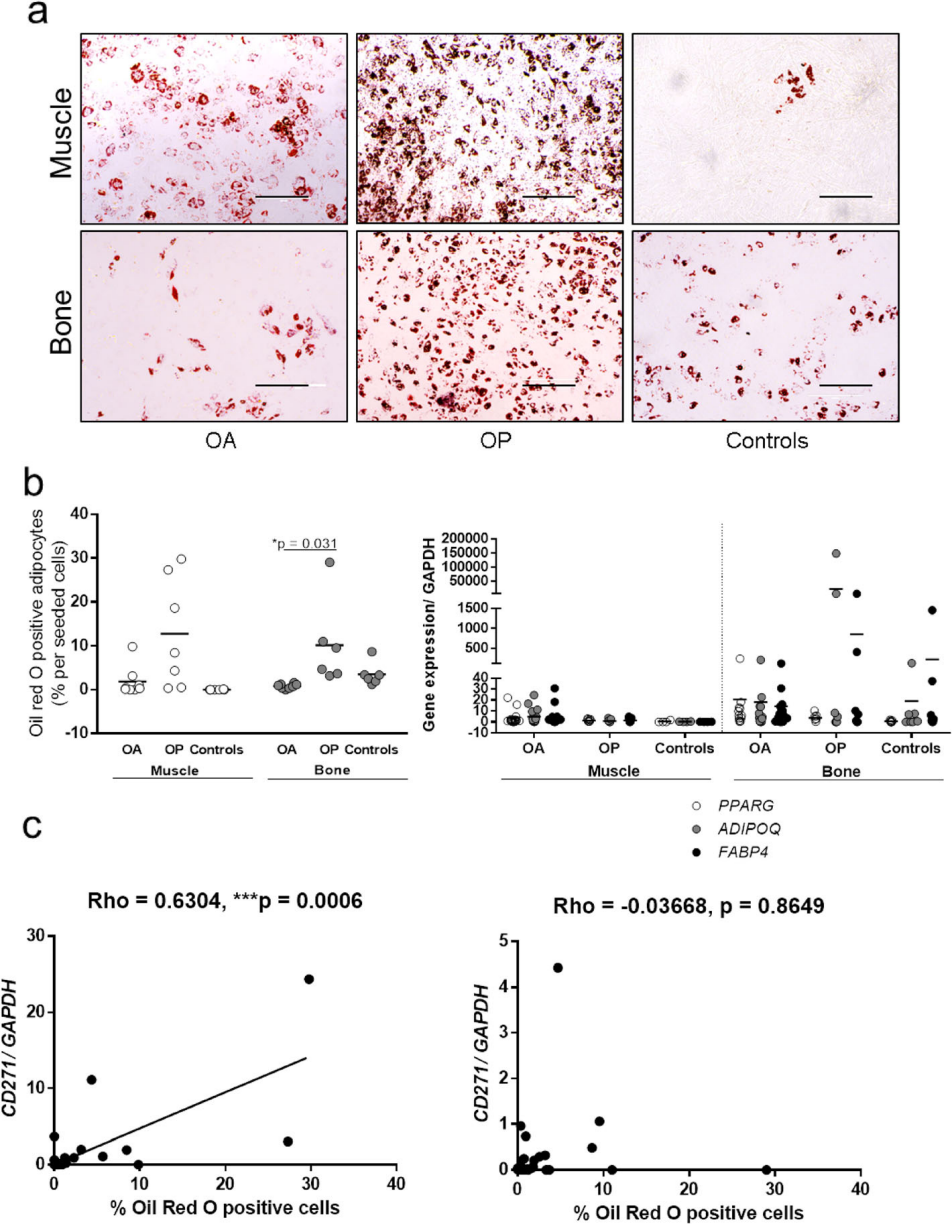
Adipogenic potential of the muscle- and bone-derived MSCs. Representative images (**a**) and quantification (**b**) from Oil Red O staining analysis for the muscle- and bone-derived MSCs. Oil Red O-positive adipocytes were counted (**b**, left) and expression of adipogenic genes was measured to assess adipogenesis (**b**, right). Bone-derived MSCs from patients with OA showed lower adipogenic potential compared to those from OP patients (0.242 and 8.726%, *p* = 0.031) (**b**, left). No significant differences were observed for the muscle-derived MSCs. No significant differences were observed for the expression of the adipogenic genes (**b**, right). Scale bars: 200 μm. PPARG, peroxisome proliferator-activated receptor-gamma; ADIPOQ, adiponectin; FABP4, fatty acid-binding protein 4. Scatter plots showing the correlation between *CD271* gene expression and proportions of Oil Red O-positive adipocytes in muscle-derived MSCs (left, Spearman correlation), and no correlations for bone-derived MSCs (right) (**c**)

Adipogenic differentiation of the muscle- and bone-derived MSCs was investigated to evaluate differences between the donor groups (Fig. 5). Bone-derived MSCs from patients with OA showed lower adipogenic potential compared to those from OP patients (Fig. 5a, b, left), as shown by the significantly lower proportion of Oil Red O-positive adipocytes (0.242 and 8.726%, *p* = 0.031). No significant differences were observed for the muscle-derived MSCs. No significant differences were observed for the expression of the adipogenic gene adiponectin, peroxisome proliferator-activated receptor gamma (PPARG), and fatty acid-binding protein 4 (FABP4) (Fig. 5b, right).

Correlation analysis showed a highly significant positive correlation between the proportion of Oil Red O-positive adipocytes and the expression of *CD271* in muscle-derived MSCs (Rho = 0.6304, *p* = 0.0006) (Fig. 5c, left), whereas bone-derived MSC showed no such correlation (Rho = − 0.03668, *p* = 0.8649) (Fig. 5c, right).

### MSCs from osteoarthritis patients have lower osteogenic and chondrogenic potentials in vitro

**Fig. 6:**
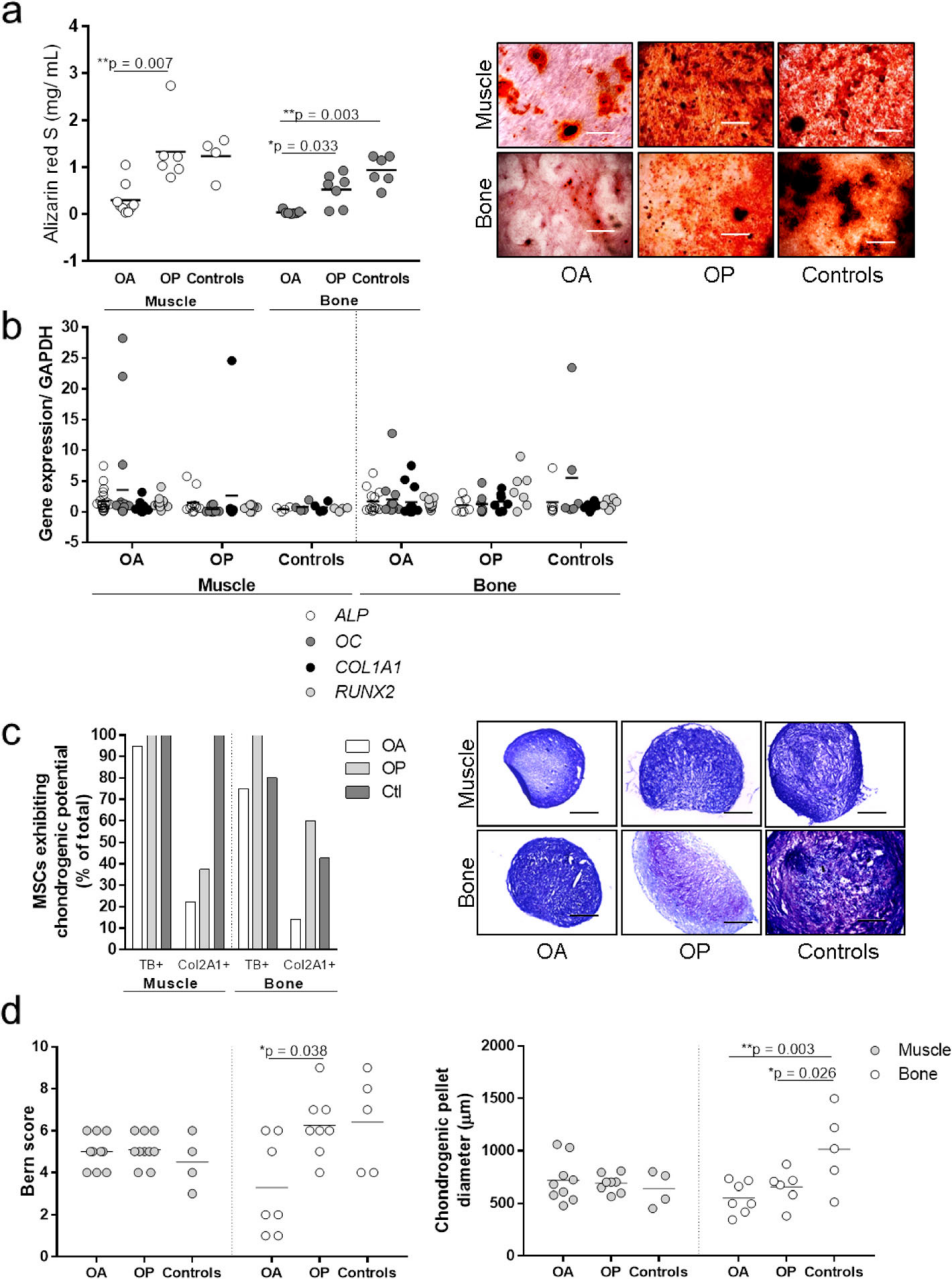
Osteogenic and chondrogenic potentials of muscle- and bone-derived MSCs. Culture-expanded cells were tested for osteogenic (**a**, **b**) and chondrogenic (**c**, **d**) potentials. Alizarin Red S staining (**a**) and osteogenic genes (**b**) were measured to assess osteogenesis. Muscle-derived MSCs from OA patients showed significantly lower Alizarin Red S concentrations (0.302 vs. 1.33 mg/mL, *p* = 0.007). Bone-derived MSCs from OA patients showed significantly lower Alizarin Red S concentrations compared to MSCs from OP patients and controls (0.04386 vs. 0.5329 and 0.9433 mg/mL, *p* = 0.033 and 0.003, respectively) (**a**). Toluidine blue and collagen type II (Col2A1) staining (**c**), Bern score (**d**, left), and pellet diameter (**d**, right) measurements were performed to assess chondrogenesis. Representative images of toluidine blue-stained chondrogenic pellets are shown (**c**, right). Scale bars: 200 μm

In addition to adipogenesis, osteogenic and chondrogenic differentiation was also examined to confirm the multipotent phenotype of the MSCs in vitro and to evaluate any functional differences between the donor groups (Fig. 6). Muscle-derived MSCs from OA patients showed significantly lower osteogenic potential compared to MSCs from OP patients, as shown by the significantly lower Alizarin Red S concentrations (Fig. 6a) (0.302 vs. 1.33 mg/mL, *p* = 0.007). Bone-derived MSCs from OA patients showed significantly lower osteogenic potential compared to MSCs from OP patients and controls, as shown by the significantly lower Alizarin Red S concentrations (Fig. 6a) (0.04386 vs. 0.5329 and 0.9433 mg/mL, *p* = 0.033 and 0.003, respectively).

However, there were no differences in the expression of the osteogenic gene alkaline phosphatase (ALP), osteocalcin (OC), alpha-1 type I collagen (COL1A1), and runt-related transcription factor 2 (RUNX2) (Fig. 6b).

Moreover, bone-derived MSCs from OA patients also showed lower chondrogenic potential compared to those from OP patients and controls, as shown by the lower proportions of toluidine blue and collagen type II (Col2A1)-positive chondrogenic pellets (Fig. 6c), the significantly lower Bern score (3.45 vs. 6.86; *p* = 0.038, Fig. 6d, left), and the significantly lower diameters of the chondrogenic pellets compared to the controls (553.3 vs. 1139.0 μm, *p* = 0.003, Fig. 6d, right). Chondrogenic pellets from OP patients also showed significantly lower diameters compared to the controls (652.8 vs. 1139.0 μm, *p* = 0.026, Fig. 6d, right).

### Bone-derived MSCs from osteoarthritis patients show lower proportions of CD73-positive cells

**Fig. 7:**
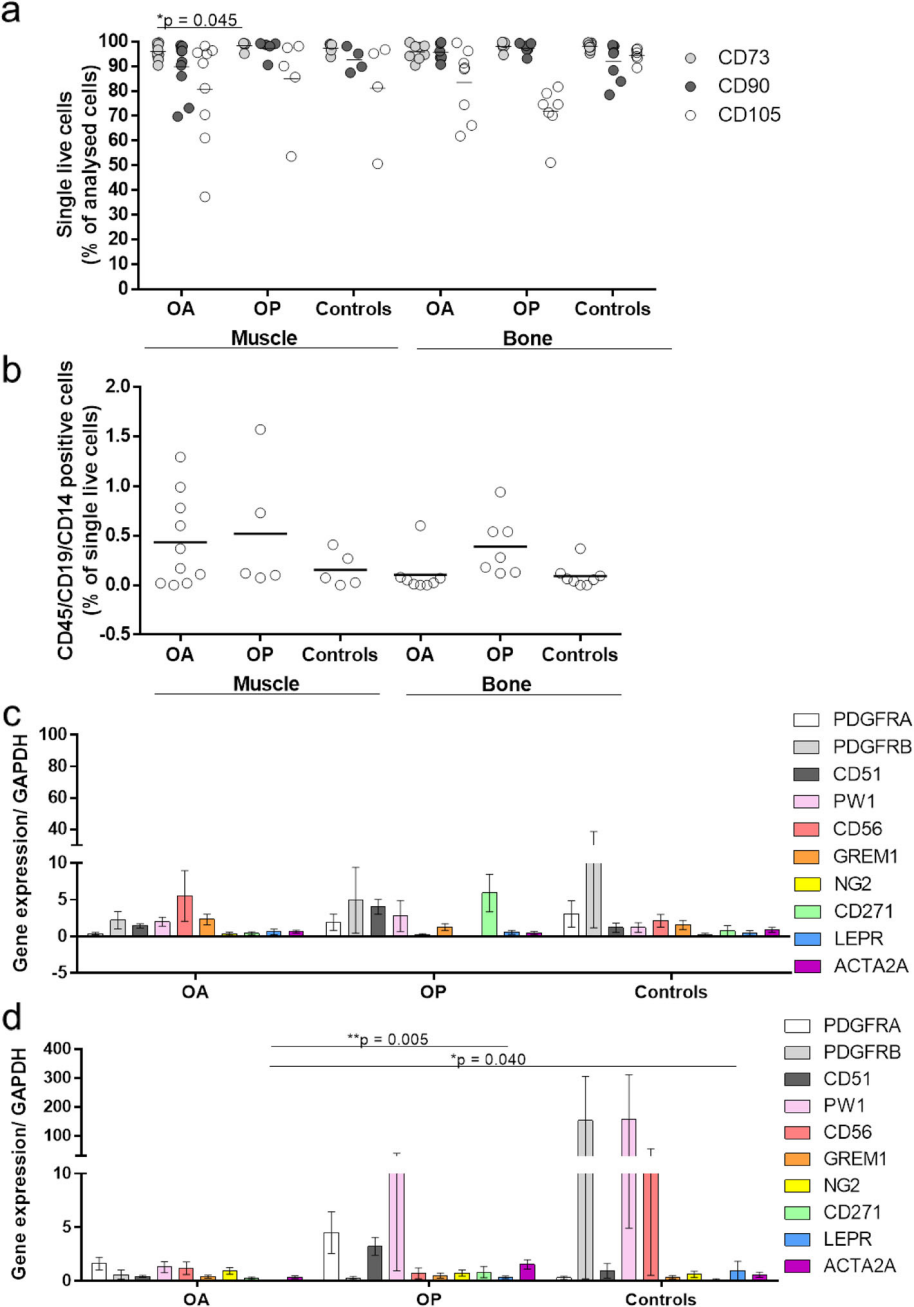
In vitro immunophenotype and gene profiling of markers of MSC subpopulations. Flow cytometry was used to determine the rate of positive (**a**) and negative (**b**) markers, according to the International Society of Cellular Therapy [25]. Gene expression profiling for MSC markers in the muscle (**c**) and bone-derived MSCs (**d**) revealed that the *LEPR* gene was significantly downregulated in OA patients in bone-derived MSCs, compared to OP patients and controls (means 0.0085 vs. 0.334 and 1.076; *p* = 0.005, 0.040, respectively) (**d**). There were no differences for the muscle-derived MSCs between the tested groups (**c**)

To further confirm that the in vitro expanded muscle- and bone-derived cells have an MSC-like phenotype, their immunophenotypes were evaluated according to the International Society of Cellular Therapy [25]. Cells from all of these donor groups showed MSC-like phenotypes, with high expression (> 95% of cells) of the MSC-positive markers CD73, CD90, and CD105 (Fig. 7a) and low expression (< 2% of cells) of the MSC-negative markers CD45, CD14, and CD19 (Fig. 7b). Only the mean for CD105 was < 95% for all of the donor groups and for both tissues (Fig. 7a). Patients with OA showed significantly lower proportions of CD73-positive cells compared to patients with OP for muscle-derived MSCs (94.7% vs. 98.3%, *p* = 0.045).

### MSCs from patients with osteoarthritis and osteoporosis show lower expression of the MSC marker leptin receptor

To determine whether there was a preferential subpopulation of these MSCs that expanded in vitro for any particular patient group, the gene expression profiles of 10 previously identified MSC subpopulation markers were compared between the MSCs from these patient groups (Fig. 7c, d). The expression levels seen for these multitest comparisons showed that the expression of the gene for leptin receptor *LEPR* was significantly lower in the bone-derived MSCs from OA patients compared to OP patients and controls (means 0.0085 vs. 0.334 and 1.076; *p* = 0.005, 0.040, respectively, Fig. 7d). There were no differences in the muscle-derived MSCs between the tested groups (Fig. 7c).

## Discussion

Stem cell exhaustion and decrease in regenerative potential have been defined as hallmarks of aging [5]. Osteoarthritis and osteoporosis are very common degenerative disorders of the elderly and affect different tissues of the musculoskeletal system. The aim of the present study was to carry out a comprehensive analysis of MSCs from two of the main musculoskeletal sources, the well-recognized bone-derived MSCs, and the less defined muscle-derived MSCs, in patients with OA and OP, and to compare these to healthy controls.

Our data show that OA patients have lower proportions of muscle-derived MSCs, as defined by the mesenchymal fractions of ex vivo primary cells. Muscle-derived cells in both groups of patients, i.e., OA and OP patients, showed higher clonogenicity when freshly isolated and seeded at p0. Moreover, the in vitro expanded MSCs from OA patients (in particular from the bone) have a lower capacity for multilineage differentiation, as shown by the poorer adipogenesis, osteogenesis, and chondrogenesis. Patients with OP also showed poorer chondrogenesis in terms of the size of the chondrogenic pellets produced in vitro from bone-derived MSCs. These properties demonstrate the exhaustion of the MSCs in these particular patients, both in terms of their proportions and properties, which imply that muscle- and bone-derived MSCs represent targets for treatments for reversion of these two age-related disorders.

Previous studies have mainly focused on the analysis of the single disorders, and in particular of OA, where it has been shown that bone marrow-derived MSCs show reduced chondrogenic potential [6, 7]; alternatively, comparison of the two cartilage disorders with different etiologies have been carried out [26]. MSCs in OP patients have been studied to a lesser extent; however, these studies have shown that bone-marrow-derived MSCs in OP patients can switch their differentiation potential from osteogenesis to adipogenesis, thus possibly contributing to the pathogenesis of OP [27]. The present study supports these previous findings for bone-derived MSCs; even though our results did not reach the statistical difference, we did observe the highest rate of adipogenesis of both bone- and muscle-derived MSCs in OP patients. Moreover, we also showed here that muscle- and bone-derived MSCs from OA patients are less osteogenic compared to those from OP patients.

Adipose tissue infiltration into the skeletal muscle contributes to the development of sarcopenia. The cause of this, however, remains unclear. The muscle contains different progenitor cells, which include the myogenic progenitors, satellite cells, PW1+ progenitor cells, and fibro-adipogenic progenitor cells (FAPs) [1]. In the healthy skeletal muscle, a balance is maintained between these myogenic progenitors and the FAPs, and the FAPs are believed to aid in muscle regeneration [28, 29]. Kozlowska et al. showed that MSCs from the healthy skeletal muscles have the limited adipogenic potential [30], and Marinkovic et al. showed that seeding purified skeletal muscle stem cells and FAPs in co-culture at a 1:1 ratio inhibits lipid droplet formation even under adipogenic conditions [31]. Aging can cause changes in skeletal muscle progenitor cells (e.g., decreased numbers, senescence). It is therefore possible that a decrease in the myogenic progenitor cells together with an inflammatory microenvironment stimulates muscle adipogenesis by different FAPs. Indeed, our data confirm that skeletal muscle-derived MSCs from healthy donors have limited adipogenesis, which is increased significantly in patients with age-related OP. We also show that there is a correlation between skeletal muscle-derived MSC adipogenesis and the expression of *CD271*. MSCs that express *CD271* have previously been associated with cartilage defects and have been found in areas of osteochondral angiogenesis [7]. They have also been associated with rheumatoid arthritis, and they are believed to have pro-inflammatory properties [32].

We also screened in vitro expanded MSCs for gene expression of several markers of MSCs that were identified previously either in bone (e.g., leptin receptor, Gremlin 1) [3, 33], cartilage (e.g., Gremlin 1) [3], or muscle (e.g., PW1, CD56, ACTA2A) [1] regeneration or in wound healing (e.g., PDGFRB) [34]. The present study shows that *LEPR* is significantly lower in patients with OA compared to patients with OP and the controls in bone-derived MSCs. Zhou et al. identified a subpopulation of bone marrow-derived MSCs, defined by leptin receptor expression, that are able to form the bone, cartilage, and adipocytes in culture and upon transplantation in vivo [33]. Leptin receptor-positive cells arise also postnatally and give rise to most bone and adipocytes in the adult bone marrow, including the bone regenerated after irradiation or fracture [33].

Our data thus indicate that the proportion of this stem cell population is lower in OA patients, which might be implicated in the pathogenesis of this particular disorder, as has been suggested previously [5].

Here, we performed a comprehensive analysis with the inclusion of well-recognized MSC sources, as the bone tissue and the less recognized skeletal muscle, to determine whether MSCs from these two tissues are affected by the common skeletal disorders of OA and OP. Another advantage of the present study is the inclusion of healthy donors, without OA or OP, who represent the controls for degenerative disorders such as OA and OP. However, the MSCs from the healthy controls were isolated from tissues obtained by these donors post mortem. It is particularly difficult to obtain tissue from healthy donors, for obvious ethical reasons. The tissues in our study were obtained on average 23 h post mortem from the same anatomical sites of the *gluteus medius* and the subchondral bone of the hip, and the MSCs were isolated and culture-expanded in the same way as those from the patients. Therefore, we believe that these donors represent the best controls possible. The donors were not age-matched, and they were significantly younger than the OP patients (Table 1), as the complete exclusion of some form of age-related OA and OP would be extremely difficult in such age-matched donors. The harvesting of the tissue sources used in this study from healthy age-matched donors would have been unethical to perform. This limitation was taken into account with the appropriate statistical analysis using age as covariate hence controlling the influence of this known factor on MSC properties. In this way, the differences in MSC properties reported here are induced by the disease progression and not by the age of the donor. Another potential limitation of this study is that the OP patients all had to have a hip fracture. Consequently, early fracture healing mechanisms might have influenced some of the parameters of the MSCs analyzed in this study. However, this is a genuine situation associated with the emergence of this particular skeletal disorder.

## Conclusions

In conclusion, we have identified changes in MSCs in the muscle and bone tissues of OA and OP patients. Whether these changes are a consequence of these two disorders or actually contribute to their pathogenesis remains to be defined potentially in functional animal models of these two disorders. The results of our study show endogenous MSC exhaustion in OA and OP patients, not only of bone-derived MSCs, but also of muscle-derived MSCs, although to a lesser extent. These findings support the need for future clinical trials to evaluate the properties of stem cell therapies in these two degenerative disorders, with the ultimate aim being the regeneration of the affected bone and muscle in these patients.

## Supplementary information

**Additional file 1: Supplementary Table S1.** Primer pair sequences used for the gene expression profiling [35–43].

## Data Availability

All data generated and/or analyzed during this study are included in this published article.

## Abbreviations

ACTA2A: Smooth muscle alpha (α)-2 actin
CFU-F: Colony-forming unit fibroblast assays
Col2: Collagen type II
DMEM: Dulbecco’s modified Eagle’s medium
GAPDH: Glyceraldehyde-3-phosphate dehydrogenase
GREM1: Gremlin 1
MSCs: Mesenchymal stem/stromal cells
p: Passage
LEPR: Leptin receptor
NG2/CSPG4: Chondroitin sulfate proteoglycan 4
OA: Osteoarthritis
OP: Osteoporosis
PDGFRα/β: Platelet-derived growth factor α/β
PW1/PEG3: Paternally expressed 3
TGF-ß1: Transforming growth factor ß1
