# Supplementary material for: Comprehensive analysis of skeletal muscle- and bone-derived mesenchymal stem/stromal cells in patients with osteoarthritis and femoral neck fracture
Source: Stem Cell Res Ther. 2020 Apr 3;11:146. doi: 10.1186/s13287-020-01657-z (PMC7118858; doi:10.1186/s13287-020-01657-z)
Supplement: Supplementary file 1 — Additional file 1: Supplementary Table S1. Primer pair sequences used for the gene expression profiling [35–43]. [file 13287_2020_1657_MOESM1_ESM.docx]

**Supplementary Table S1.** Primer pair sequences used for the gene expression profiling.

| **Gene symbol** | **Gene name** | **Forward primer (5'-3')** | **Reverse primer (3'-5')** | **Reference** |
| --- | --- | --- | --- | --- |
| *ADIPOQ* | Adiponectin, C1Q and collagen domain containing | TGGTGAGAAGGGTGAGAA | AGATCTTGGTAAAGCGAATG | [35] |
| *ALP* | Alkaline phosphatase | CCAAGTACTGGCGAGACCAA | GTGGAGACACCCATCCCATC | Our laboratory |
| *COL1A1* | Collagen type I alpha 1 | GCCAAGACGAAGACATCCCA | GTTTCCACACGTCTCGGTCA | Our laboratory |
| *CD51* | Integrin alpha V | GGTCCCCAAGTCACTCCAAG | GATTCATCCCGCAGATACGC | Our laboratory |
| *CD56* | Neural cell adhesion molecule 1 | GTCCTGCTCCTGGTGGTTGT | TGACCGCAATGCACATGAA | [36] |
| *CD271* | Nerve growth factor receptor | ACCTCCAGAACAAGACCTCATAGC | TTGTTCTGCTTGCAGCTGTTCC | [37] |
| *FABP4* | FABP4 fatty acid binding protein 4 | TGGTTGATTTTCCATCCCAT | GCCAGGAATTTGACGAAGTC | [38] |
| *GAPDH* | Glyceraldehyde-3-phosphate dehydrogenase | TGCACCACCAACTGCTTAGC | TGGCATGGACTGTGGTCATG | [39] |
| *GREM1* | Gremlin 1 | AACAGTCGCACCATCATCAA | AATTTCTTGGGCTTGCAGAA | [40] |
| *LEPR* | Leptin receptor | GTGAAGCCTGATCCACCATT | CCCCTCACCTGAACCTCATA | [41] |
| *NG2* | Chondroitin sulfate proteoglycan 4 | AGAACAAAGGTCTCTGGGTCC | TGTGACCTGGAAGAGCACAT | Our laboratory |
| *BGLAP* | Bone gamma-carboxyglutamate protein | AGCGAGGTAGTGAAGAGAC | GAAAGCCGATGTGGTCAG | [41] |
| *PDGFRA* | Platelet derived growth factor receptor alpha | GGTGGTCACAGGTGCTTTTT | AAACCACTTAAGGCTCTCAGGA | [42] |
| *PDGFRB* | Platelet derived growth factor receptor beta | TGAGAAAGATCGAGATTGTGCG | GGGCTTCGGGTCACAGG | [42] |
| *PPARG* | Pperoxisome proliferator activated receptor gamma | CACAAGAACAGATCCAGTGGTTGCAG | AATAATAAGGTGGAGATGCAGGCTCC | [37] |
| *PW1* | Paternally expressed 3 | GCCTAGCCCCACCTTTAGTG | CTTACACACCCTGCACTCGT | Our laboratory |
| *RUNX2* | Runt related transcription factor 2 | AGCAAGGTTCAACGATCTGAGAT | TTTGTGAAGACGGTTATGGTCAA | [43] |
